# Normal body mass index (BMI) can rule out metabolic syndrome: An Israeli cohort study: Erratum

**DOI:** 10.1097/MD.0000000000015744

**Published:** 2019-05-13

In the article, “Normal body mass index (BMI) can rule out metabolic syndrome: An Israeli cohort study”,^[1]^ which appears in Volume 98, Issue 9 of *Medicine*, the authors’ first and last names were reversed. The authors’ names should appear as Ofer Kobo, Ronit Leiba, Ophir Avizohar and Amir Karban.
